# A Qualitative Assessment of Patient Experience following Systematic Implementation of Goals of Care Conversations in the Ambulatory Gynecologic Oncology Setting

**DOI:** 10.1089/pmr.2022.0040

**Published:** 2022-11-22

**Authors:** Nicole C. Zanolli, Luke A. Gatta, Laura Fish, Margaret Falkovic, Amelia Lorenzo, Allison M. Puechl, Laura J. Havrilesky, Brittany Davidson

**Affiliations:** ^1^ Department of Obstetrics and Gynecology, Duke University School of Medicine, Durham, North Carolina, USA.; ^2^ Department of Obstetrics and Gynecology, Duke University Medical Center, Durham, North Carolina, USA.; ^3^ Duke Cancer Institute Behavioral Health and Survey Research Core, Durham, North Carolina, USA.; ^4^ Department of Population Health Sciences, Durham, North Carolina, USA.; ^5^ Division of Gynecologic Oncology, Duke University Medical Center, Durham, North Carolina, USA.

**Keywords:** communication, end of life, goals of care, gynecologic cancers, patient–clinician relationship

## Abstract

**Objective:**

Although skilled goals of care (GOC) conversations are known to reduce aggressive futile end-of-life care, they have not been widely implemented nor standardized in the care of gynecologic malignancies. Clinicians express concern regarding patient readiness and willingness to participate in these conversations, which may be a barrier to GOC discussions.

**Methods:**

This is a qualitative study, conducted at an academic institution in the United States, of patients with gynecologic malignancies at high risk of death within six months and who had recently completed a GOC discussion with their oncology clinician during an ambulatory visit. Within 10 days of this conversation, patients were approached for potential participation in an hour-long semistructured interview. Patients enrolled in hospice or who were non-English speaking were excluded. Participants were enrolled until thematic saturation was reached. Interviews were transcribed and coded using the five-stage thematic approach.

**Results:**

Ten women were consented and participated in semistructured interviews, which occurred a median of 4 (range 1–18) days after the index GOC discussion. The median age was 64 (range 37–78), and the most common diagnosis (50%) was recurrent platinum-resistant ovarian cancer. Four themes were identified: (1) delivery of the GOC conversation, (2) importance of prioritizing individual values, (3) involving family in decision making, and (4) openness to discussing discontinuation of anticancer treatment and hospice. Patients generally felt these GOC conversations were useful, providing a space to express their values and did not compromise the patient–clinician relationship.

**Conclusions:**

Patients seemed willing to engage in GOC conversations and were appreciative of their clinicians' communication skills. Often, they used this conversation as an opportunity to convey personal values affecting their care.

## Introduction

Patients with cancer often receive aggressive treatment at the end of life (EOL) despite extensive evidence demonstrating higher health care costs, excessive resource utilization, and worse quality of life without survival benefits.^1–3^ As such, aggressive EOL treatment is commonly considered a marker of poor quality cancer care.^3–6^ Specifically, goals of care (GOC) conversations conducted >30 days before death are associated with lower incidences of chemotherapy in the last 14 days of life, hospitalization and intensive care unit (ICU) admission in the last month of life, dying in an acute care setting, and admission to hospice less than three days before death.^5^

GOC conversations should be more than conversations about code status. These individualized discussions between patients, their families, and their health care team allow for opportunities to revisit prognosis, elucidate an individual's values and goals for their care, and subsequently allow for a goal-concordant treatment recommendation from the clinician.^1,7^ Despite evidence supporting the benefits of early and outpatient GOC discussions, many barriers exist in integrating these conversations into clinical care, including perceived lack of clinician time, clinician fear of patient readiness for these conversations, and lack of clinician communication training and proficiency.^8^

Patients with gynecologic cancers are a unique population as they are traditionally cared for by a single clinician, who provides both surgical intervention and medical management of their cancer throughout the duration of their cancer trajectory. Although this has many benefits it may also present a barrier as clinicians with long-term relationships with their patients may hesitate to initiate these challenging conversations for fear of degrading trust or rapport.^9,10^

In a retrospective 2013 study at our institution, more than one-third of gynecologic oncology patients participated in their first documented GOC discussion during their final hospital admission, usually during their last month of life.^5^ In addition, 20% of patients did not have a single documented GOC discussion before death, identifying a gap in our then current practice pattern.^5^

In August 2017, we implemented a successful quality improvement initiative at our academic institution to systematically implement a framework for early, outpatient GOC discussions to address this deficiency, which has been reported elsewhere.^11^ We hoped that the systematic implementation of GOC conversation would enhance the clinician–patient relationship and allow for the receipt of goal-concordant cancer care. The focus of this qualitative study is to explore the patient experience following earlier outpatient GOC conversations.

## Materials and Methods

**FIG. 1.**
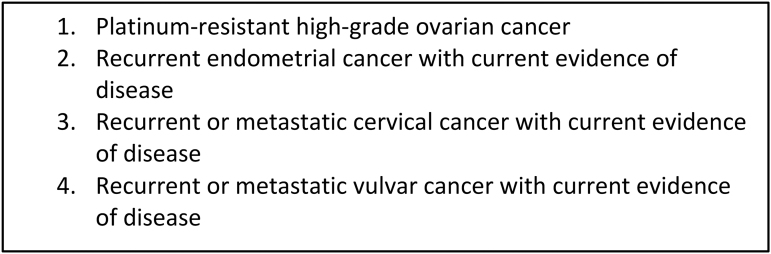
Clinical diagnoses defined as high risk of death within six months.

This study was approved by the Institutional Review Board (Pro00092891). In August 2017, a quality improvement project to systematically implement outpatient GOC conversations at a single academic institution in North Carolina was initiated. Gynecologic oncology patients at high risk for death in six months were systematically and prospectively identified based on their current disease status and in accordance with clinical criteria (Fig. 1).

**FIG. 2.**
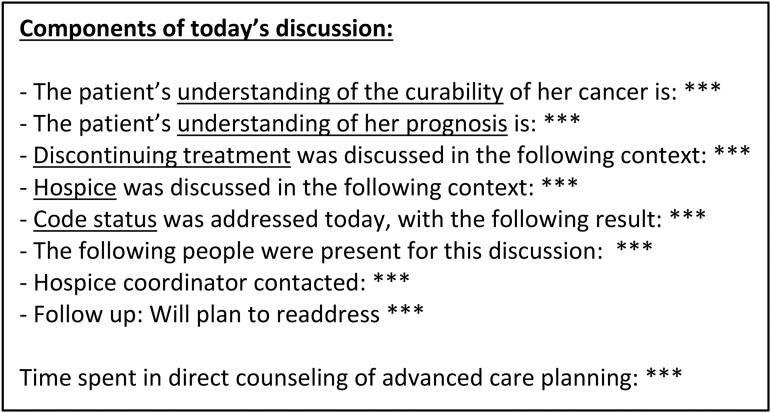
EMR template for GOC conversation. EMR, electronic medical record; GOC, goals of care.

For the purposes of this study, “high-risk” patients were identified by trained nursing staff and the patient's primary gynecologic oncologist was prompted to initiate a GOC conversation within three outpatient gynecologic oncology visits. Suggested content of the GOC discussion was integrated within an electronic medical record template for ease of use and standardization of documentation (Fig. 2). GOC discussions could be addressed in a variety of contexts, including discussion of scan results, recent hospitalization, or declining functional status.

Patients eligible for this qualitative study included English-speaking patients at least 18 years of age with an outpatient GOC discussion in the last three weeks that was documented using the electronic medical record (EMR) template. Exclusion criteria included patients with current hospice enrollment or those who participated in a GOC conversation during an inpatient admission. Inpatient and hospice patients were excluded as this study focused on patients participating in GOC conversations in outpatient settings. Non-English-speaking patients were excluded given that Spanish-language consents were not available. We purposefully sampled eligible patients by race, partner status, and cancer diagnosis to ensure a breadth of perspective. Participating subjects received $50 for their time.

Informed consent was obtained from all participants. Basic demographics and cancer data were abstracted from the medical record (L.A.G. and N.C.Z.). Experienced qualitative researchers from the institution's Behavioral Health and Survey Research Core completed the semistructured hour-long interviews (M.F. and L.F.). An interview guide was used to guide conversations (Supplementary Material S1). Recruitment continued until thematic saturation was reached. Audio recordings were created with encrypted devices and transcripts were downloaded using AtlasTi 7.5 software (Berlin, Germany). A five-staged approach was implemented to conduct thematic analysis (familiarization, identifying a thematic framework, indexing, charting and mapping, and interpretation). The entire study team reviewed the initial two to three transcripts to develop the coding framework. All transcripts were then coded by two independent study team members.

## Results

Ten subjects were consented for participation between October 2018 and May 2019. Although no formal screening log was kept during the duration of the study, ∼20 patients were approached to recruit our study cohort. Baseline demographics are demonstrated in Table 1. The median age of participants was 64 (range 37–78); four (40%) of participants were Black and six (60%) were White. The most common diagnosis (50%) was recurrent platinum-resistant ovarian cancer. The median duration of patient/clinician relationship before the GOC conversation was 26.4 months (4.7–169.7) An attending physician initiated and conducted all GOC conversations.

**Table 1.** Patient Demographics

Median age, years (range)	64 (37–78)
Race	*n* (%)
Black	4 (40)
White	6 (60)
Ethnicity	
Hispanic	0 (0)
Non-Hispanic	10 (100)
Marital status	
Partnered	6 (60)
Unpartnered: single, divorced, or widowed	4 (40)
Recurrent cancer diagnosis	
Cervical	1 (10)
Endometrial	3 (30)
Ovarian	5 (50)
Vulvar	1 (10)
ECOG status before GOC conversation	
1	5 (50)
2	2 (20)
3	3 (30)
On active treatment when GOC conversation occurred	
No	4 (40)
Yes	6 (60)
No. of prior treatments	
0	1 (10)
1	1 (10)
2	0 (0)
3	6 (60)
4+	2 (20)
Median length of patient/clinician relationship, months (range)	26.4 Months (4.7–169.7)
Median length of time between GOC conversation and study interview, days (range)	4 Days (1–18)

ECOG, Eastern Cooperative Oncology Group; GOC, goals of care.

The majority (80%) of the participants were interviewed by phone. The median time between the index GOC conversation and the interview was four days (range 1–18).

Four major themes were independently identified by two coders: (1) delivery of the GOC conversation, (2) importance of prioritizing individual values, (3) involving family in decision making, and (4) openness to discussing discontinuation of anticancer treatment and hospice. Quotations from participants highlighting each theme are displayed in Table 2.

**Table 2.** Thematic Organization of Representative Participant Quotations During Goals of Care Discussions

Theme	Quote
Delivery of the GOC conversation	
Delivery	“[They have] a way of delivering bad news in a good way.”—63-year-old participant with ovarian cancer “Having it put very gently made a really big difference.… [They] didn't dash all my hopes of survival, but [they were] realistic about the expectations you know, expect the worst but hope for the best.”—37-year-old participant with cervical cancer “Yet [they were] gentle in [their] wording you know, [they] would touch me, [they] would reach out and put [their] hand on my hand when I started crying and you know, would be like, okay get tissues you know, and [they] just, it was very compassionate and I felt like I was dealing with someone that saw my pain and felt sorry you know, they felt bad about it.”—37-year-old participant with cervical cancer “But [they] come in there and [they're] just so you know, no emotion type person you know what I'm saying? And so, I mean [they] says what [they have] to say and that's about it.”—48-year-old participant with ovarian cancer
Individual voice	“[They] get that my quality of life is very important, so we're gonna do everything we can to let me be normal and garden and do things with my kids and keep me out of pain so that I'm not being able to function.”—37-year-old participant with cervical cancer “I think that [they] understand, I really do. [They've] tried very hard to find different types of treatment for me. I feel like that [they] clearly understand what's going on with me and [they're] a very compassionate doctor. Knows that I like to, go out and have lunch with my girlfriends and things like that. [They] know that.” -70-year-old participant with ovarian cancer
Honesty	“Most of all that [they were] going to be honest with me. That was really critical to me, that when you know in your mind that the gig is up, I want to know that and no fancy words.”—77-year-old participant with ovarian cancer “Dr. [name] has been straightforward with me. [They] didn't beat around any bushes from the day I met [them] you know, and you know, a lot of doctors they tell you something to see if you'll accept that and then they don't go any further in the conversation.”—55-year-old participant with vulvar cancer “I think [they were] honest and I appreciate that.”—64-year-old participant with uterine cancer
Importance of prioritizing individual values	
Relationships with others	“My main thing…I want to live long enough to see my granddaughter grow up…I need to fight this”—55-year-old participant with vulvar cancer
Specific hobbies/leisurely activities	“I went whitewater rafting over in [state]. I went up into the mountains there by [place], at the falls, and went down slippery rock falls twice. I mean yeah, I did things that I never thought that I would get to do, and I had a wonderful time.”—77-year-old participant with ovarian cancer “I mean I couldn't walk because the blisters were bad and I finally got those healed up and I did finally go [to California on a hike].”—78-year-old participant with ovarian cancer
Side effect/pain tolerability	“I'm not ready to leave this earth but I know my time is coming on down and all I've ever said is you all keep me comfortable. When I get to the point when I am sick, sick and can't do anything, keep me comfortable.”—78-year-old participant with ovarian cancer “[My doctors] have made me feel very comfortable…and you know which is really, really important to me.”—77-year-old participant with ovarian cancer
Treatment options	“I wanted to fight this no matter what. You know, put me through trials, let me be a guinea pig. I'll do whatever.”—37-year-old participant with cervical cancer “I've been fighting all this time and I'm gonna fight till the end.”—56-year-old participant with uterine cancer “I didn't want to get back on anything, any chemo or anything.”—70-year-old participant with ovarian cancer
Involving family in decision making	
Barrier to GOC conversation	“I initially was fairly open to having my children there but then they wanted to interject too much. So, I ended up a lot of the time really wanting to see [them] alone because there was just too much of [my children] being intrusive. I think back even four or five months ago before I started the second chemo, and [my children] just seemed like this push, push, push toward chemo and you know… I told [them] you know, I'm feeling good, I'm going to movies, I'm going out to lunch, I'm walking with my neighbor you know, I mean I'm taking care of my house. [My daughter] is coming at it from one place and I'm coming from another place and I don't seem to be able to make [them] you know, understand.”—77-year-old participant with ovarian cancer
Openness to discussing discontinuation of anticancer treatment and hospice	
Quality versus quantity	“Yeah, we backed off. I looked down and I said can I have some quality rather quantity? And they agreed with me.”—78-year-old participant with ovarian cancer “You know, I'd rather have the two months of good time, than the four months of, I'm miserable”—66-year-old participant with endometrial cancer
Trust in clinician	“I told [them] I trusted [them] to let me know when it reached the point where I would be at, where I could still back out gracefully so to speak.”—77-year-old participant with ovarian cancer “Well, I completely and totally trust Dr. [name] and especially [name], because I've been connected with them for so long.”—66-year-old-participant with uterine cancer “I said, I need to be able to trust you and [they] said, you'll be able to.”—77-year-old participant with ovarian cancer
Timing of hospice	“I never meant that in the sense that anytime I feel bad I should be throwing in the towel. I said, I'm sick from chemo. I said, most everybody gets sick or has something go on when they're being treated for cancer. I said, what did you think, it was a free ride? [My daughter] kept thinking oh, I'm really sick you know, within that week after chemo, and so therefore I should be going to hospice and so we argued that point from a year ago.”—77-year-old participant with ovarian cancer “I knew that when you go on hospice, you really cannot do anything and for me to choose hospice like, right now, would be me cutting off a little bit of life that I have left with my family…And so, to me, hospice is not something that I'm thinking about a lot because I think by the time I will be in hospice, it won't matter to me at all.”—63-year-old participant with ovarian cancer

### Delivery of the GOC conversation

Participants emphasized the importance of the technique of content delivery, feeling as if their clinician heard and understood them, and the importance of honesty throughout each of the 10 interviews. Most participants (8 of 10) discussed how the style of delivery of the GOC conversations impacted their experience with their clinician. The majority of participants felt that the GOC conversations were appropriately delivered. A few identified opportunities for improvement in the clinician's delivery, namely inclusion of empathic statements throughout the conversation. These sentiments were reflected with statements such as the following:

Yet [they were] gentle in [their] wording you know, [they] would touch me, [they] would reach out and put [their] hand on my hand when I started crying and you know, would be like, okay get tissues you know, and [they] just, it was very compassionate and I felt like I was dealing with someone that saw my pain and felt sorry you know, they felt bad about it.—37-year-old participant with cervical cancer

Most participants reported that the GOC conversation allowed the opportunity for their clinician to hear and understand them as individuals. In addition, several participants discussed the importance of honesty from their clinician during GOC conversations with statements such as the following:

Most of all that [they were] going to be honest with me. That was really critical to me, that when you know in your mind that the gig is up, I want to know that and no fancy words.—77-year-old participant with ovarian cancer

### Importance of prioritizing individual values

During the interview, participants were asked to share the content of their GOC conversation with their clinician. In all 10 interviews, the importance of prioritizing ones' individual values was highlighted. Within this discussion, the following four values were highlighted as priorities: (1) relationships with others, (2) specific hobbies/leisurely activities, (3) side effect/pain tolerability, and (4) treatment options. In addition, patients readily proposed prioritizing their time for leisurely activities or completion of “bucket list” items.

### Involving family in decision making

Each participant was asked the questions “Who came with you to your appointment?” and “After the appointment, did you talk with family or close friends about the GOC conversation? Why or why not?” Only 1 out of the 10 patients did not have any family participate in the GOC conversation. Responses to these questions were divided into two groups: (1) patients who regarded their families as integral and active participants in their GOC conversations and decisions and (2) patients who regarded their families as well-intentioned although holding discordant opinions in matters of GOC. Specifically, four patients noted that their family dynamics were often a barrier during GOC conversations. In each of the four cases, the family members were inclined to pursue ongoing treatment and the patients themselves intended to focus primarily on symptom management.

### Openness to discussing discontinuation of anticancer treatment and hospice

Most participants acknowledged that although discussing discontinuation of anticancer treatment and/or hospice was a challenging conversation, they felt favorably about how the discussion was approached and handled by their clinician. Participants often highlighted the importance of quality versus quantity of time. In addition, trust in their clinician to let them know when it was time to stop treatment and start hospice was a common theme and is reflected by statements such as the following:

I told [them] I trusted [them] to let me know when it reached the point where I would be at, where I could still back out gracefully so to speak.—77-year-old participant with ovarian cancer

Although most participants trusted their clinician to provide guidance on discontinuing anticancer treatment and initiating hospice, some responses reflect confusion surrounding the appropriate timing of hospice, with some participants feeling rushed into hospice, whereas others preferring to delay hospice enrollment until death was imminent.

## Discussion

Prior research demonstrates that clinician communication through GOC discussions is associated with better quality of life and positive caregiver experiences, reduces use of futile medical care, and is cost-effective.^5,12–14^ The findings of this qualitative study reinforced the notion that clinician–patient communication is an essential component in the treatment of patients with gynecologic malignancies, particularly near the EOL. Participants discussed four themes: qualities of the GOC conversation, prioritizing values, involving family in decision making, and discussing discontinuation of anticancer treatment and hospice.

Despite data demonstrating numerous benefits of GOC discussions in the care of patients with cancer, these conversations are neither standard nor widely adapted. In one study, only 14% of women with ovarian cancer recalled a structured GOC conversation as part of their care.^15^ GOC conversations vary in their detail, with some limited to a discussion of patient's code status, whereas others are a more nuanced narrative entry of a patient's knowledge of their disease, prognosis, and elicitation of goals with subsequent goal-concordant treatment recommendation. Despite both quantitative and qualitative evidence supporting GOC discussions, barriers exist in integrating these conversations into clinical care, including the perception of lack of clinician time and communication skills training and clinician fear of patient nonreadiness for these conversations.

Although the sample size of our study was small, this qualitative study addresses the latter point and reinforced prior study that patients are willing to engage in these conversations, are appreciative of their clinicians' communication efforts, and may have concerns regarding the involvement of family members in these discussions. Our data support explicit questioning of the patient regarding who they want to participate (or not participate) in these discussions to ensure patient comfort and candidness.

In addition, patients reported differing opinions on the appropriate timing of hospice care. The potential for patient misconception of what hospice care entails is important for providers to be aware of and plan for. This confusion could represent a knowledge gap that may be a target for future interventions, potentially increasing patient comfort, knowledge base, and willingness to engage in GOC discussions earlier in the disease process.

In a multicenter survey of 1256 clinicians caring for seriously ill patients, “family members' or patients' difficulty accepting a poor prognosis” was identified as the single most important barrier to initiating GOC conversation.^8^ Owing to the perceived gravity of the conversation, clinicians may fear patient willingness and readiness to participate in a GOC, especially when discussions may focus on prognosis, offers to discontinue cancer-directed treatment, and pursue hospice care. There are several themes coded in this study that suggest the opposite. None of the patients in this study expressed doubt about the utility or need for this conversation.

Prioritizing values is foundational to medical decision making and the delivery of goal-concordant care. With the patient able to identify and express their values, the clinician is then given the opportunity to recommend treatments that are in line with those wishes and, most importantly, forego therapies that may pose a barrier to attaining the individual's goals. These qualitative findings are consistent with previous study in patients with nongynecologic malignancies. In one prior qualitative study of patients with advanced cancer (Stage 4 or Stage 3 unresectable lung, pancreatic, or biliary cancer), patients viewed GOC conversation as an important tool to maintain a trusting relationship with their clinician.^16^

Several questions still remain, including the timing of GOC conversations and the specific content discussed. Our study adds to existing evidence refuting the concern that discussing EOL topics contributes to patient distress and supports a wider implementation of GOC conversations for patients with gynecologic malignancies.^12^

Our study has several limitations. As participants had to agree to take part in an interview, participation bias is inevitable. Subjects who chose to participate in the study may have been more reflective or willing to discuss EOL topics compared with those who declined participation. In addition, some eligible patients were not well enough to be interviewed, limiting the generalizability of the study. Finally, the study population included only English speakers, which introduces the potential for cultural bias if patients from non-English-speaking backgrounds and those who require use of an interpreter have different openness to GOC conversations.

A strength of this study was the clinical timeframe for enrollment. Patients were eligible only if a GOC conversation occurred within three weeks of their scheduled study interview, intended to limit the effect of recall bias. In addition, interviews were conducted among participants representing a diverse set of gynecologic malignancies, whereas the previous literature has focused primarily on patients with ovarian cancer.

## Conclusion

Our study demonstrates that high-risk patients with gynecologic malignancies near the EOL were not only receptive to GOC discussions with their health care team, but also identified these as opportunities to share their values. The conversations helped highlight the importance of trust between clinicians and patients and did not appear to compromise this relationship. Efforts aimed to improve clinician communication skills could also further improve effectiveness of GOC conversations, as patients felt the style of delivery of the GOC conversations, as well as clinician understanding, impacted their overall experience.

## Supplementary Material

Supplemental data

## Abbreviations Used

ECOG: Eastern Cooperative Oncology Group
EMR: electronic medical record
EOL: end of life
GOC: goals of care
ICU: intensive care unit
