# Supplementary material for: A Qualitative Assessment of Patient Experience following Systematic Implementation of Goals of Care Conversations in the Ambulatory Gynecologic Oncology Setting
Source: Palliat Med Rep. 2022 Nov 22;3(1):308–15. doi: 10.1089/pmr.2022.0040 (PMC9712054; doi:10.1089/pmr.2022.0040)
Supplement: Supplemental data [file Suppl_MaterialS1.docx]

**Supplemental Material 1. Interview Guide**

Research project title: Ambulatory goals of care conversation in high-risk gynecologic oncology patients: a qualitative assessment of patient experience

Interviewer: Thank you for agreeing to take part in this project to improve care at the Duke Cancer Institute. I will go through the consent form with you. Review consent form with the patient Interviewer: I am interested in hearing your thoughts and reactions to the appointment you had with Dr. ___ on ___.

1. Before your clinic visit, what did you understand about your diagnosis? What did you understand about your treatment options?

2. During the appointment on ___, what, if anything, did you learn about your diagnosis and your treatment plan?

3. During your appointment, Dr. ___ asked you specific questions about your goals for treatment. Please tell me what you recall from this conversation. Probe for thoughts and feelings

4. When Dr. ___ asked about your goals for treatment, what did you share? Probe for values

5. Who came with you to the appointment? Is that usual? Why or why not?

6. After the appointment, what did you discuss with friends and family?

7. How did your conversation change the way you think about the care you’re getting from the medical team?

8. How much do you feel that your doctor understood what was important to you? Probe for thoughts 9. How much do you feel that your doctor made a recommendation that matched what you said you wanted?

10. Did the conversation you have change your feelings of trust in your doctor and your team? Why or why not? How satisfied were you with the goals of care conversation?

11. We know that it is important to understand patient goals and values when caring for them. From your perspective, what could your doctor do to improve this type of discussion?
